# Pamphlets Received

**Published:** 1858-09

**Authors:** 


*Pamphlets Received.*—We have been compelled to do injustice to the authors of a number of interesting essays which we have received, in not giving reviews or analyses of their contents; but our original department has been so overflowing, that such reviews have been necessarily excluded. In despair of ever being able to give more extended notices, we mention a few that have been lately received:

*A Report on Diseases of the Cervix Uteri. Read before the Medical Society of the State of Georgia, at the Annual Meeting in Augusta, April,* 1857. By JOSEPH A. EVE, M. D., Prof. of Obstetrics and Diseases of Women and Children in the Medical College of Georgia.

We received this essay some time ago, as the date indicates, and expected to be able to give it a more extended notice than we can now give. The author takes the only rational view in regard to the use of the speculum; the charge of indelicacy which is brought against it so ardently, will apply as well to a multitude of operations which are absolutely indispensable. The only question is, is it a valuable means of diagnosis? and that it is so, we conceive is now generally conceded.

*Dr. Paine's Essays on Vitality and Remedial Agents.* 1842.

We tender our acknowledgments to the author for this valuable essay; the doctrines of which are discussed in the review of "*Paine's Institute of Medicine*" in this number.

*Lectures on the Sulphate of Quinia. Delivered in the Regular Course of the Medical Department of the University of Michigan.* By A. B. PALMER, A. M., M. D., Prof. of Materia Medica, Therapeutics and Diseases of Women and Children. Published by the Class.

This is a pamphlet of sixty pages, containing four lectures, delivered before the students of the medical department of the University of Michigan. The subject is treated in an able and masterly manner, and does credit both to the author and the appreciation of the class who desired their publication. We cannot, however, agree with Dr. Palmer as to the necessity of preparatory treatment in the administration of quinia in malarious fevers; we have seen the best effects produced by its immediate administration, and are confident that preparatory treatment is entirely unnecessary.

*Pathology and Treatment of the Paralysis of Motion.* By J. P. BATCHELDER, M. D., New York.

The author reports eleven cases which he treated at the New York Hospital, all of which were considerably improved, and five entirely cured. Dr. Batchelder lays great stress upon judicious and regular exercise, which he has always found to be of benefit.

We have also received "A Valedictory Address to the Medical Graduates of Harvard University," by Prof. OLIVER WENDELL HOLMES, M. D. This is written in the pleasing familiar style which is so characteristic of its author, and inculcates some most admirable lessons for the young physician.

An Essay on Prolapsus of the Funis, by T. GALLIARD THOMAS, M. D. This is a paper read before the New York Academy of Medicine, in which the author recommends the postural treatment for falling down of the cord. He places his patients on their knees with the thorax depressed, precisely reversing the direction of the inclined plane of the uterus. It is certainly reasonable to suppose that, in this position, the cord would be retained when returned within the uterus. Dr. Thomas supports his views by two cases successfully treated.

We would also express our thanks to the authors, for a copy of "The Anatomy of the Placenta, by John C. Dalton, M. D., Prof. of Physiology and Microscopical Anatomy in the College of Physicians and Surgeons of New York," which is extracted from the American Medical Monthly, and "Medical Opinion in the Parish Will Case, by Pliny Earle, M. D., formerly Physician to the Bloomingdale Asylum for the Insane." These papers we will probably discuss in a future number of the Journal; now we have space merely to mention them.

---

*The Medical Journal of North Carolina.*—This is the title of a new Journal which has been started in North Carolina, edited by Edward Warren, M. D. It is a bi-monthly of a hundred pages, and presents a creditable appearance. The first number is a good one, and with the resources which North Carolina must have, the Journal will be well sustained and will succeed. We welcome it most cordially.

---

*Resignations and Appointments.*—Dr. J. B. McClellan has been elected to fill the vacancy in the Pennsylvania Medical College, occasioned by the removal of Dr. Richardson to New Orleans.

Dr. S. G. Armor has resigned the chair of Pathology and Clinical Medicine in the Missouri Medical College, and Dr. McMartin, of St. Louis, has been appointed to fill the vacancy.

Dr. Austin W. Nichols having been compelled, by ill health, to resign the lectureship of Descriptive Anatomy, in the medical department of the University of Buffalo, Dr. Sandford Eastman, of this city, has been elected by the Faculty, to fill the vacancy. Dr. Eastman will bring to the duties of the chair, zeal and industry, and we have no doubt will render himself most acceptable as a lecturer, and in all the relations of teacher and pupil.

